# 47 Burn Injury Assessment Study (BIAS): Is There Room To Improve?

**DOI:** 10.1093/jbcr/irad045.021

**Published:** 2023-05-15

**Authors:** Jeffrey Carter, Herbert Phelan, John Friedstat, William Hickerson, James Holmes, James Hwang, Steven Kahn, Alisa Savetamal, Jeffrey Shupp

**Affiliations:** University Medical Center New Orleans & LSUHSC, River Ridge, Louisiana; University Medical Center- New Orleans Burn Center, New Orleans, Louisiana; MGH, Boston, Massachusetts; Access Pro Medical, LLC, Augusta, Georgia; Wake Forest School of Medicine, Winston-Salem, North Carolina; UAB, Birmingham, Alabama; The South Carolina Burn Center/Medical University of South Carolina, Charleston, South Carolina; Yale New Haven Health, Bridgeport Hospital, Bridgeport, Connecticut; Firefighters' Burn and Surgical Research Laboratory, MedStar Health Research Institute, Georgetown University School of Medicine, Washington, District of Columbia; University Medical Center New Orleans & LSUHSC, River Ridge, Louisiana; University Medical Center- New Orleans Burn Center, New Orleans, Louisiana; MGH, Boston, Massachusetts; Access Pro Medical, LLC, Augusta, Georgia; Wake Forest School of Medicine, Winston-Salem, North Carolina; UAB, Birmingham, Alabama; The South Carolina Burn Center/Medical University of South Carolina, Charleston, South Carolina; Yale New Haven Health, Bridgeport Hospital, Bridgeport, Connecticut; Firefighters' Burn and Surgical Research Laboratory, MedStar Health Research Institute, Georgetown University School of Medicine, Washington, District of Columbia; University Medical Center New Orleans & LSUHSC, River Ridge, Louisiana; University Medical Center- New Orleans Burn Center, New Orleans, Louisiana; MGH, Boston, Massachusetts; Access Pro Medical, LLC, Augusta, Georgia; Wake Forest School of Medicine, Winston-Salem, North Carolina; UAB, Birmingham, Alabama; The South Carolina Burn Center/Medical University of South Carolina, Charleston, South Carolina; Yale New Haven Health, Bridgeport Hospital, Bridgeport, Connecticut; Firefighters' Burn and Surgical Research Laboratory, MedStar Health Research Institute, Georgetown University School of Medicine, Washington, District of Columbia; University Medical Center New Orleans & LSUHSC, River Ridge, Louisiana; University Medical Center- New Orleans Burn Center, New Orleans, Louisiana; MGH, Boston, Massachusetts; Access Pro Medical, LLC, Augusta, Georgia; Wake Forest School of Medicine, Winston-Salem, North Carolina; UAB, Birmingham, Alabama; The South Carolina Burn Center/Medical University of South Carolina, Charleston, South Carolina; Yale New Haven Health, Bridgeport Hospital, Bridgeport, Connecticut; Firefighters' Burn and Surgical Research Laboratory, MedStar Health Research Institute, Georgetown University School of Medicine, Washington, District of Columbia; University Medical Center New Orleans & LSUHSC, River Ridge, Louisiana; University Medical Center- New Orleans Burn Center, New Orleans, Louisiana; MGH, Boston, Massachusetts; Access Pro Medical, LLC, Augusta, Georgia; Wake Forest School of Medicine, Winston-Salem, North Carolina; UAB, Birmingham, Alabama; The South Carolina Burn Center/Medical University of South Carolina, Charleston, South Carolina; Yale New Haven Health, Bridgeport Hospital, Bridgeport, Connecticut; Firefighters' Burn and Surgical Research Laboratory, MedStar Health Research Institute, Georgetown University School of Medicine, Washington, District of Columbia; University Medical Center New Orleans & LSUHSC, River Ridge, Louisiana; University Medical Center- New Orleans Burn Center, New Orleans, Louisiana; MGH, Boston, Massachusetts; Access Pro Medical, LLC, Augusta, Georgia; Wake Forest School of Medicine, Winston-Salem, North Carolina; UAB, Birmingham, Alabama; The South Carolina Burn Center/Medical University of South Carolina, Charleston, South Carolina; Yale New Haven Health, Bridgeport Hospital, Bridgeport, Connecticut; Firefighters' Burn and Surgical Research Laboratory, MedStar Health Research Institute, Georgetown University School of Medicine, Washington, District of Columbia; University Medical Center New Orleans & LSUHSC, River Ridge, Louisiana; University Medical Center- New Orleans Burn Center, New Orleans, Louisiana; MGH, Boston, Massachusetts; Access Pro Medical, LLC, Augusta, Georgia; Wake Forest School of Medicine, Winston-Salem, North Carolina; UAB, Birmingham, Alabama; The South Carolina Burn Center/Medical University of South Carolina, Charleston, South Carolina; Yale New Haven Health, Bridgeport Hospital, Bridgeport, Connecticut; Firefighters' Burn and Surgical Research Laboratory, MedStar Health Research Institute, Georgetown University School of Medicine, Washington, District of Columbia; University Medical Center New Orleans & LSUHSC, River Ridge, Louisiana; University Medical Center- New Orleans Burn Center, New Orleans, Louisiana; MGH, Boston, Massachusetts; Access Pro Medical, LLC, Augusta, Georgia; Wake Forest School of Medicine, Winston-Salem, North Carolina; UAB, Birmingham, Alabama; The South Carolina Burn Center/Medical University of South Carolina, Charleston, South Carolina; Yale New Haven Health, Bridgeport Hospital, Bridgeport, Connecticut; Firefighters' Burn and Surgical Research Laboratory, MedStar Health Research Institute, Georgetown University School of Medicine, Washington, District of Columbia; University Medical Center New Orleans & LSUHSC, River Ridge, Louisiana; University Medical Center- New Orleans Burn Center, New Orleans, Louisiana; MGH, Boston, Massachusetts; Access Pro Medical, LLC, Augusta, Georgia; Wake Forest School of Medicine, Winston-Salem, North Carolina; UAB, Birmingham, Alabama; The South Carolina Burn Center/Medical University of South Carolina, Charleston, South Carolina; Yale New Haven Health, Bridgeport Hospital, Bridgeport, Connecticut; Firefighters' Burn and Surgical Research Laboratory, MedStar Health Research Institute, Georgetown University School of Medicine, Washington, District of Columbia

## Abstract

**Introduction:**

The treatment of burn injured patients requires accurate wound assessment to determine appropriate interventions. Clinical assessment of burn depth, even by experts in the field, has an accuracy of only 60–80%. The purpose of this study is to estimate the performance of burn wound assessment by burn care professionals from still images.

**Methods:**

This was an IRB-approved prospective cohort study in which burn care providers used a tablet-based digital interface to answer a 10-item questionnaire and to identify non-healing burn wounds from 5 patients scenarios. Each wound was evaluated using a polygon software interface to mark non-healing regions within the wound. Each wound had a ground truth determined by a consensus panel of study investigators using still photograph at 21-days post-injury to discern healing and non-healing regions. A power analysis and sample size of 100 participants produced a two-sided 95% confidence interval with a width equal to 0.20 when the sample proportion is 0.60. Continuous variables were analyzed by mean, standard deviations, median and quartiles while categorical data was analyzed as exact number and percentage. Burn wound assessment performance metrics for the providers were calculated for the whole cohort and then for subgroups using point estimate at a pixel level. Accuracy, sensitivity, specificity, area positive predictive value (APPV), and area negative predictive value (ANPV) were calculated from the ground truth and participants selected regions of non-healing wounds.

**Results:**

One hundred fifty-six healthcare providers enrolled (50 physicians, 14 APPs, 63 nurses, 24 therapists, and 5 paramedics) with 9.96 ± 9.49 mean years of experience and 70% employed at a verified burn center. All five regions were represented in the analysis (Eastern Great Lakes (4%), Midwestern (6.5%), Northeastern (22%), Southern (53.5%), and Western (14%). 29.3% of the participants selected “schedule for surgery” when reviewing images of wounds that healed without surgery. 28.7% of the participants selected, “local wound care no surgery needed or, “local wound care, reassess in 7+ days for possible surgery.” Performance measures were not associated with years of experience. Overall performance is available in Table 1.

**Conclusions:**

Improving burn wound assessment performance could improve our use of limited resources in burn care by optimizing patient transfers/treatments and avoiding unnecessary surgeries. Our study expands upon prior work in the field and demonstrates that burn wound assessment has significant room for improvement. These findings open the proverbial door for diagnostic devices found in other fields of medicine and our partnership with emergency room providers to advance burn care.

**Applicability of Research to Practice:**

More accurate wound assessment will lead to improved treatment and triage by EM and Burn practitioners.

